# Controlling Morphology and Aggregation in Semiconducting Polymers: The Role of Solvents on Lasing Emission in Poly[2-methoxy-5-(2′-ethyl-hexyloxy)-1,4-phenylene-vinylene]

**DOI:** 10.3390/ma10070706

**Published:** 2017-06-29

**Authors:** Minghuan Liu, Yonggang Liu, Zenghui Peng, Chengliang Yang, Quanquan Mu, Zhaoliang Cao, Ji Ma, Li Xuan

**Affiliations:** 1 State Key Laboratory of Applied Optics, Changchun Institute of Optics, Fine Mechanics and Physics, Chinese Academy of Sciences, Changchun 130033, China; liuminghuan13@mails.ucas.ac.cn (M.L.); liuyonggang@ciomp.ac.cn (Y.L.); peng@ciomp.ac.cn (Z.P.); ycldahai@ciomp.ac.cn (C.Y.); muquanquan@ciomp.ac.cn (Q.M.); caozlok@ciomp.ac.cn (Z.C.); jma2@kent.edu (J.M.); 2 University of Chinese Academy of Sciences, Beijing 100049, China

**Keywords:** semiconducting polymer, holographic polymer dispersed liquid crystal, aggregation and morphology, amplified spontaneous emission, polymer dispersed liquid crystal

## Abstract

Systematic experiments were performed to investigate solvent-dependent morphology and aggregation of the semiconducting polymer film poly[2-methoxy-5-(2′-ethyl-hexyloxy)-1,4-phenylene-vinylene] (MEH-PPV), which was span-cast from nonaromatic strong polarity solvents tetrahydrofuran (THF), trichloromethane (TCM) and aromatic weak polarity solvents chlorobenzene (CB), toluene, and p-xylene. The results indicated that the conformation of the spin-cast MEH-PPV films with weak aggregation such as THF and TCM demonstrated excellent lasing emission performances because of inhibiting the fluorescence quenching induced by bi-molecule process. The Atomic Force Microscope (AFM) images confirmed the distinct morphologies of the spin-cast MEH-PPV films. The amplified spontaneous emission (ASE) was investigated in a simple asymmetric slab planar waveguide structure by methods of variable stripe length (VSL) and shifting excitation stripe (SES). The amplified spontaneous emission (ASE) experiments confirmed the distinct polymer chain conformation. The conformation, which preserved from the spin-cast process, indicated the distinct interactions between solvents and MEH-PPV polymer chains. The pure film spectra were performed to confirm the effect of distinct conformation on the material energy level. This work provides insights into the morphology and aggregation effect of the spin-cast polymer films on the performances of lasers.

## 1. Introduction

Organic solid-state lasers (OSSLs) have attracted more attention recently [1,2,3]. Many materials and device configurations has been developed in this area. Semiconducting materials like semiconducting polymers are promising due to high efficiency, wide spectral coverage, and solution-based processing [4,5]. There have been no demonstrations of commercial devices under electrical injection using semiconducting polymers as active materials, largely because the presence of both injected polarons and metal electrodes quenches the luminescence and raises the threshold [1]. Fortunately, the indirect electrical pumping using inexpensive light emitting diode (LED) is feasible [6]. Therefore, the OSSLs based on semiconducting polymers can be potentially used in sensors, optical communications, and integrated photonics [6,7,8].

Despite the versatility for photonic applications, some of the fundamental physical insights underlying the fabrication or optimization of practical devices based on those organic semiconducting materials remain poorly understood. The dissolution environment of the semiconducting polymers plays a vital role on the morphologies, packing conformation of the casting films [9,10,11] and electroluminescent performance [11,12], because the aggregation conformation in solvents will preserve the spin-cast films. The semiconducting polymer aggregation is a place where two or more chain segments come together and share their *π*-electron density [11]. The microstructure of solution processed thin films has been investigated by X-ray diffraction. The results demonstrate that the poly [2-methoxy-5-(2′-ethyl-hexyloxy)-1,4-phenylene-vinylene] (MEH-PPV) backbones and the planes defined by the benzene rings within the phenylene vinylene (PPV) backbones are predominantly parallel to the film plane [13]. The nanoscopic inter-chain aggregation domain formation is observed by the combination of third harmonic generation (THG) and near-field scanning optical microscopy (NSOM) [14]. Researchers Lampert et al. report the dependence of optical gain to solvents [15]. There are numerous limit reports on lasing emission of the semiconducting polymer films spin-cast from different solvents in a slab planar waveguide structure. Implement such a study will create a physical insight into the effect of solvent-dependent film morphology and aggregation on the lasing emission of spin-cast semiconducting materials.

In this study, the morphology and aggregation of the spin-cast semiconducting polymer, poly[2-methoxy-5-(2′-ethyl-hexyloxy)-1,4-phenylene-vinylene] (MEH-PPV) films was systematically studied in nonaromatic strong polarity solvents tetrahydrofuran (THF), trichloromethane (TCM) and aromatic weak polarity solvents chlorobenzene (CB), toluene and p-xylene. The morphologies were investigated and imaged with an Atomic Force Microscope (AFM). The amplified spontaneous emission (ASE) was investigated in a simple asymmetric slab planar waveguide structure. In the waveguide structure, it contained a MEH-PPV layer as the core layer and a polymer-dispersed liquid crystal (PDLC) film/pre-clean glass substrate as the cladding layers. The net gain and waveguide losses of the spin-cast MEH-PPV films were also investigated and compared. The spectra of the pure films were performed. In the last section, the lasing emission of the spin-cast MEH-PPV films in a distributed feedback (DFB) configuration based on holographic polymer dispersed liquid crystal (HPDLC) were characterized to reveal the practical application.

## 2. Materials and Methods

### 2.1. Semiconducting Layer Preparation

The MEH-PPV film was used as an active medium. The MEH-PPV (OLED Material Tech.) was dissolved in tetrahydrofuran (THF), trichloromethane (TCM), chlorobenzene (CB), toluene and p-xylene by weight ratio at 0.6 wt %. The chemical structures and properties of five solvents used in this study are shown in Table 1. The solutions were stirred for 48 h to ensure sufficient dissolution. A drop of MEH-PPV solution was injected onto a piece of deionized pre-clean glass substrate for spin-cast. The thickness of the MEH-PPV film was controlled by the spin speed and measured using a surface profiler (KLA Tencor P-16+). All experiments were performed in air under the same ambient circumstance.

### 2.2. Waveguide Structure Fabrication

**Figure 1:**
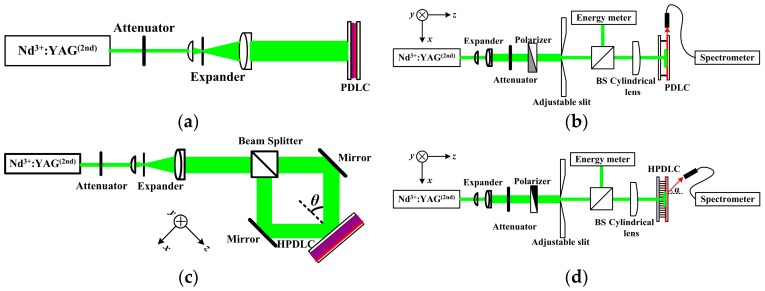
Experimental setups: (**a**) Schematic presentation of the experimental setup for polymer-dispersed liquid crystal (PDLC) film fabrication; (**b**) diagram of poly[2-methoxy-5-(2′-ethyl-hexyloxy)-1,4-phenylene-vinylene] (MEH-PPV) film amplified spontaneous emission (ASE) pumping; (**c**) experimental setup of holographic polymer dispersed liquid crystal (HPDLC) laser fabrication with a two-beam Mach-Zehnder interferometer, and; (**d**) HPDLC distributed feedback (DFB) laser optical performance characterization schematic diagram.

In order to characterize the amplified spontaneous emission (ASE) properties of MEH-PPV film spin-cast from different solvents, the polymer dispersed liquid crystal (PDLC)/MEH-PPV/glass substrate slab planar waveguide structure was fabricated. The PDLC film was fabricated on the MEH-PPV film as a cladding layer by photochemical reaction. The mixture for PDLC mainly contained acrylate monomers dipentaerythritol hydroxyl pentaacrylate (DHPA, Aldrich, 29.4 wt %, Shanghai, China) and phthalicdiglycol diacrylate (PDDA, Eastern Acrylic Chem, 29.4 wt %, Shandong, China) and nematic liquid crystals (TEB-30A, *n_o_* = 1.522, Δ*n* = 0.170, Silichem, 29.4 wt %, Shijiazhuang, China). Crosslinking monomer N-vinylpyrrolidone (NVP, Aldrich, 9.8 wt %, Shanghai, China) was also added to dilute the mixture. Rose Bengal (RB, Aldrich, 0.5 wt %, Shanghai, China) and N-phenylglycine (NPG, Aldrich, 1.5 wt %, Shanghai, China) were used as photoinitiator and coinitiator, respectively. The mixture, which was stirred for 48 h to ensure an isotropic and homogeneous material system, was injected into an empty glass cell by capillary action in a darkroom. The empty sample cell was made by two pieces of glass substrates, one had a spin-cast MEH-PPV film and the other was a pure glass substrate. The cell gap was controlled at 9 μm by spacers. The PDLC film was photo-cured by illuminating the sample for 10 min using a 532 nm continuous frequency doubled Neodymμμium-doped Yttrium Aluminum Garnet (Nd^3+^:YAG, second harmonic generation [16]) laser beam (New Industries Optoelectronics, Changchun, China) at 10 mW/cm^2^, as shown in Figure 1a. An attenuator was inserted to the beam path to regulate the beam intensity. The 2 mm initial beam diameter was expanded to 10 mm when it passed through the expander, which contained a 20-X micro objective (Newport) and a 100 mm focal length doublet lens. A pinhole (25 μm, Newport), which located at the focal plane of the micro objective, was used as a spatial filter in the expander. The mean refractive index of the PDLC after photo-polymerization was 1.541 at 589 nm, which was measured using an Abbe refractometer (2 WA, Kernco, El Paso, TX, USA).

### 2.3. ASE Characterization

For the ASE study, the samples, which had a MEH-PPV film sandwiched between a PDLC film and a glass substrate, were optically pumped by a frequency doubled passively Q-switched Nd^3+^:YAG pulsed laser (532 nm, 10 ns, 10 Hz, New Industries Optoelectronics, Changchun, China), as shown in Figure 1b. The pumping laser beams along the sample normal were reshaped with a cylinder lens (f = 200 mm) to produce a nearly rectangle pumping area on the sample. An adjustable slit was used to filter the central part (3 mm by 1 mm) of the pumping area to ensure uniform pumping. The ASE emission from the sample edge of the waveguide was then collected using a fiber-coupled grating spectrometer (Sofn Instruments, Beijing, China) with a resolution at 0.23 nm. Moreover, a non-polarized beam splitter (BS) was used in the beam path to split part of the incident beams to monitor the real time pump beam energy. An optical attenuator was used to regulate the pump energy continuously to investigate output-emission intensity as a function of input-pumping energy fluence.

**Figure 2:**
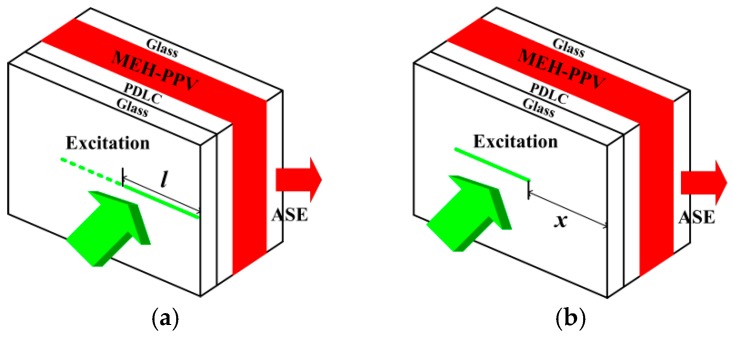
Schematic illustration for (**a**) variable stripe length and (**b**) shifting excitation stripe experiments.

The net gain of the film was obtained using a variable stripe length (VSL) method [17]. One end of the excitation stripe was in same position with the edge of the sample while the length of excitation stripe (l) was varied. The intensity of the ASE from the edge of the sample was measured, as shown in Figure 2a. The output ASE intensity should be governed by

(1) $$I(\lambda )=\frac{A(\lambda )I_{p}}{g(\lambda )}[e^{g(\lambda )l}-1],$$

where *A*(*λ*) is a constant related to the spontaneous emission cross section, *I_p_* is the pumping intensity, *g*(*λ*) is the net gain coefficient, and *l* is the length of the pumping stripe.

The waveguide losses of the MEH-PPV film were characterized as shown in Figure 2b, where the excitation stripe was gradually shifted away from the edge of the sample when keeping the excitation stripe length constant [17]. The ASE intensity from the end of the excitation stripe (*I*_0_) should be constant since the excitation energy of the pumping beam is constant. The emission from the edge of the sample decreased because the excitation stripe was shifted and the absorption/scattering loss would be increased with shifting. The waveguide losses follow the Beer-Lambert law

(2) $$I=I_{0}e^{-\alpha x},$$

where *x* is the shifting distance between the end of the excitation stripe and the edge of the sample, *α* is the waveguide losses, *I* is the ASE emission intensity from the edge of the sample, and *I*_0_ is the ASE emission intensity from the end of excitation stripe.

### 2.4. Laser Fabrication and Characterization

As for waveguide distributed feedback (DFB) laser application and characterization, a holographic polymer-dispersed liquid crystal (HPDLC) [18] film was fabricated on the MEH-PPV film as an external distributed feedback layer [19]. The prepolymerization mixture was the same with the one used for PDLC. The interference optical field was created by two laser beams to exposure the prepolymer mixture to fabricate HPDLC film, as shown in Figure 1c. A non-polarized beam splitter was used to split the incident laser beams by intensity with a ratio at 1:1 (3.05 mW/cm^2^). The curing time was controlled at 60 s with laser beams. Therefore, the glass/MEH-PPV/HPDLC waveguide structure was formed for the waveguide HPDLC DFB laser.

As shown in Figure 1d, the glass/MEH-PPV/HPDLC was optically excited for DFB laser application. A polarizer was used to regulate the polarization state of the pumping laser. The output lasing emission was collected with a fiber-coupled grating spectrometer with ~32° to the sample normal [20]. A 50 mm focal length lens was used to collect the emission light into the probe of a high resolution energy meter (Coherent) for conversion efficiency measurement.

## 3. Results and Discussion

### 3.1. Film Morphologies

**Figure 3:**
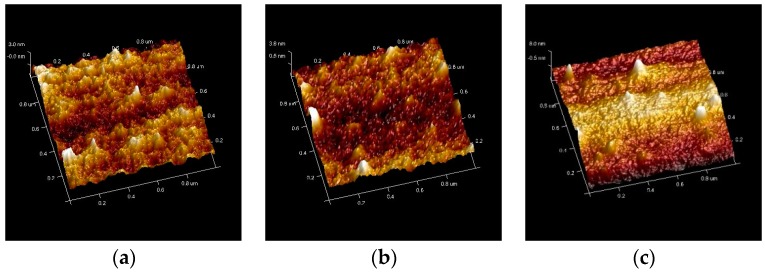

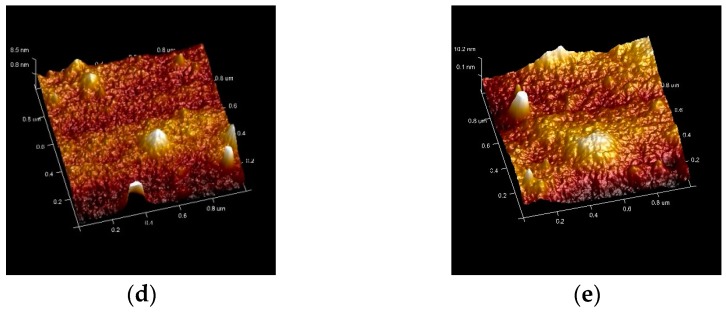
Atomic Force Microscope (AFM) image of the MEH-PPV films from (**a**) THF; (**b**) TCM; (**c**) CB; (**d**) toluene and (**e**) p-xylene.

The interactions between solvents and MEH-PPV chains vary intensely when MEH-PPV is dissolved in different solvents [11]. Therefore, the morphology conformation, which indicates the interactions between the solvents and the polymer chains, is preserved during the spin-cast process [21]. The thickness of the films was controlled at 80 nm. The tapping mode Atomic Force Microscope (AFM, BRUKER Multimode 8) was used to visually compare the film morphologies at a scan rate of 5 μm/s. Figure 3 shows the film morphologies of the MEH-PPV films spin-cast from different solvents. The average surface roughness was 0.673 nm, 0.716 nm, 1.31 nm, 1.82 nm, and 2.02 nm for THF, TCM, CB, toluene, and p-xylene spin-cast films, respectively. The results showed that the surface morphology of the MEH-PPV films changed significantly with the spin-cast solvents. The morphologies for nonaromatic strong polarity solvents THF and TCM were more flat and uniform than that spin-cast by aromatic weak polarity solvents CB, toluene and p-xylene, as shown in Table 1. It implied that the conformation of MEH-PPV chains in solution preserved through the casting process and survived into the film. Aromatic weak polarity solvents such as CB, toluene and p-xylene possess a preferential interaction with the aromatic backbone of the polymer chains, as a result, the polymer chains adopt a rigid, open conformation in solution. The nonaromatic strong polarity solvents such as THF and TCM, on the other hand, possess a preferential interaction with the polymers side groups. Thus, the polymer chains in nonaromatic strong polarity solvents tend to coil tightly to maximize solvent-side group interactions and minimize exposure of the aromatic backbone to the solvent.

The light scattering experiments show that the average hydrodynamic radius *R_H_* in aromatic weak polarity solvent CB is nearly double than that in nonaromatic strong polarity solvent THF. The difference of average hydrodynamic radius *R_H_* confirms the distinct conformation for MEH-PPV polymer chains in solutions [22]. The conformations of MEH-PPV chains in solutions will preserve the MEH-PPV films when spin-cast. The microstructures of the MEH-PPV films cast from aromatic solvents CB and p-xylene and nonaromatic solvent THF are investigated by X-ray diffraction. The results indicate that the chain packings and orientations are different. When MEH-PPV films are span-cast from THF, the anisotropy in chain orientation is more pronounced [13]. In next section, the film ASE characterization will be performed to investigate the optical performance differences of the MEH-PPV films spin-cast from different solvents.

### 3.2. Film ASE Characterization

The film morphologies are the macroscopic indication of the inner orientation conformation of the polymer chains. Thus, the ASE experiments were performed to prove the distinct inner conformation [23]. The glass/MEH-PPV/PDLC waveguide structure is a simple cavity geometry for spectral selection even though they cannot suppress the spectral well. There is a cutoff film thickness (*h_cutoff_*) in this structure [24], below which the fundamental mode cannot propagate. This cutoff thickness is given by

(3) $$h_{cutoff}=\frac{\lambda_{ASE}}{2\pi \sqrt{n_{MEH-PPV}^{2}-n_{PDLC}^{2}}}\arctan\sqrt{\frac{n_{PDLC}^{2}-n_{glass}^{2}}{n_{MEH-PPV}^{2}-n_{PDLC}^{2}}},$$

where *λ_ASE_* is central wavelength of the guided light, *n_MEH-PPV_* is the refractive index of the MEH-PPV film, *n_PDLC_* is the refractive index of the PDLC film and *n_glass_* are the refractive index of the cover glass. In our case, *λ_ASE_* is 633 nm, *n_PDLC_* is 1.541, and *n_glass_* is 1.516. The in-plane index of MEH-PPV films varied with the cast solvents. The in-plane refractive index was lower for nonaromatic solvents cast MEH-PPV films than that cast from aromatic solvents. The in-plane index was 1.94, 1.948, 1.958, 1.969 and 1.974 for THF, TCM, CB, toluene and p-xylene cast MEH-PPV films, respectively. The refractive index was ~1.53 in the direction perpendicular to the plane of the film [25]. For the spin-cast MEH-PPV, the polymer chains lie preferentially in the plane of the film [26], so we use ~1.9 in our case. The calculated cutoff thickness for MEH-PPV film was 23.9 nm. We chose the film thickness at 80 nm, which was thick enough to support the guided light. The guided light eigenvalue equation of TE-modes in a slab waveguide is described [27]:

(4) $${\left(n_{MEH-PPV}^{2}-N^{2}\right)}^{\frac{1}{2}}k_{0}h=m\pi +\arctan{\left(\frac{N^{2}-n_{PDLC}^{2}}{n_{MEH-PPV}^{2}-N^{2}}\right)}^{\frac{1}{2}}+\arctan{\left(\frac{N^{2}-n_{glass}^{2}}{n_{MEH-PPV}^{2}-N^{2}}\right)}^{\frac{1}{2}},$$

where *N* is the effective refractive index of the waveguide modes. Waveguide modeling indicated that there was only one TE-mode for an 80 nm thick film and there were no TM-modes.

**Figure 4:**
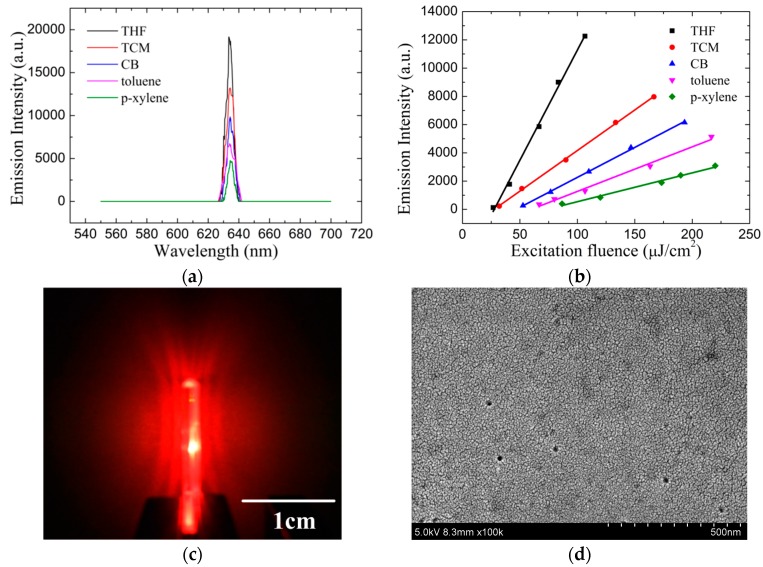
The MEH-PPV Film amplified spontaneous emission (ASE) characterization: (**a**) ASE spectra of MEH-PPV films in waveguide structure at an excitation fluence of 300 μJ/cm^2^; (**b**) the dependence of emission-pulse intensity to excitation fluence; (**c**) ASE emission pattern, and; (**d**) scanning electron microscope (SEM) image of the PDLC film.

During the photo-pumping for ASE, only the gain-narrowed peak survived and the long tails of the photoluminescence were totally compressed, when the gain over the waveguide losses. Figure 4a shows the ASE spectra of MEH-PPV films spin-cast from THF, TCM, CB, toluene and p-xylene in the waveguide structure. The ASE spectra centered at ~633 nm and the full width at half maximum (FWHM) of the spectra varied from 4.9 to 7 nm. Figure 4b shows the emission-pulse intensity as a function of excitation fluence at the central peak (633 nm). The output intensity increased with the increase in the excitation energy fluence. The THF-cast film possessed the minimum ASE threshold at 26.7 μJ/cm^2^, while the p-xylene cast film possessed the maximum ASE threshold at 86.7 μJ/cm^2^. The ASE threshold of TCM, CB, and toluene-cast film was 32, 52.7, and 66.7 μJ/cm^2^, respectively. The nonaromatic strong polarity solvents, THF and TCM, demonstrated better ASE performances than the aromatic weak polarity solvents, CB, toluene and p-xylene. For aromatic solvents, CB-cast MEH-PPV film showed better ASE performance than toluene and p-xylene. For non-aromatic solvents, THF cast MEH-PPV film showed better ASE performance than TCM. This indicates that the ASE threshold decrease with solvents molecule flatness for both non-aromatic and aromatic solvents spin-cast MEH-PPV films as shown in Table 1. The remarkable differences between the highest and lowest ASE threshold clearly shows that the solvent molecular structure and polarity induces polymer conformations distinctly. The ASE emission pattern in Figure 4c,d shows a scanning electron microscope (SEM, Hitachi S-4800) image of a PDLC film, the uniform and flat morphology confirms the excellent advantages using PDLC as the waveguide cladding layer.

### 3.3. Optical Gain and Losses

**Figure 5:**
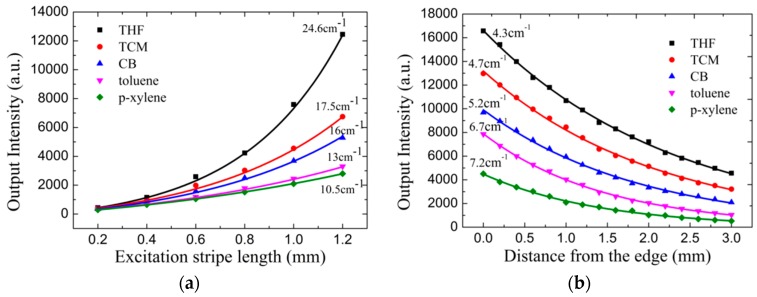
(**a**) Dependence of the film edge light intensity on the excitation stripe length with excitation fluence at 333 μJ/cm^2^. The solid lines are fitting to the data using Equation (1). (**b**) The intensity of light emitted from the edge of the waveguide as a function of the distance between the pump stripe end and the film edge at 333 μJ/cm^2^ excitation fluence. The solid lines are exponentially fitted by Equation (2).

More experiments were performed to investigate the distinct conformations of the spin-cast MEH-PPV films, which were determined by the distinct solvents polymer chains interactions. Gain and losses are intimate parameters related to the ASE in a waveguide structure [28]. Figure 5a is the ASE intensity at the central wavelength of ~633 nm dependent on the excitation stripe length with excitation fluence at 333 μJ/cm^2^ for gain study. The tendency of the fitting lines from Equation (1) were changed from steep to gentle for different solvent-cast MEH-PPV films. The fitting net gain parameters of the MEH-PPV spin-cast films from THF, TCM, CB, toluene and p-xylene were 24.6, 17.5, 16.0, 13, and 10.5 cm^−1^, as shown in Figure 5a, respectively. The nonaromatic strong polarity solvents, THF and TCM, possessed larger net gain than the aromatic weak polarity solvents, CB, toluene and p-xylene. For aromatic solvents, CB-cast MEH-PPV film possessed larger net gain than toluene and p-xylene, while for nonaromatic solvents, THF cast MEH-PPV film possessed a larger net gain than TCM. The net-gain tends to increase with the solvent molecule flatness for both non-aromatic and aromatic solvents spin-cast MEH-PPV films.

Figure 5b is the ASE intensity of light emitted at the central wavelength of ~633 nm as a function of the distance from sample edges with excitation fluence at 333 μJ/cm^2^. The fitting waveguide losses parameters of the MEH-PPV films from THF, TCM, CB, toluene and p-xylene were 4.3, 4.7, 5.2, 6.7 and 7.2 cm^−1^, as shown in Figure 5b, respectively. Again, the nonaromatic strong polarity solvents, THF and TCM, possessed smaller waveguide losses than the aromatic weak polarity solvents, CB, toluene and p-xylene. For aromatic solvents, CB cast MEH-PPV film possessed the smallest waveguide losses than toluene and p-xylene, while for non-aromatic solvents, THF cast MEH-PPV film possessed the smallest net gain for TCM. The losses tend to decrease with solvent molecule flatness for both non-aromatic and aromatic solvents spin-cast MEH-PPV films. It was showed that the surface roughness was different for MEH-PPV films spin-cast from different solvents. Therefore, it is not surprising that the losses varied with aromatic and nonaromatic solvent-cast MEH-PPV films. The rough surface affects the quality of the thin film waveguide and contributes to more scatterings when the light is guiding in the MEH-PPV films. The intense aggregation domains formed in aromatic solvent-cast MEH-PPV films can also contributed to scatterings [14]. In Section 3.4, it showed that the ground absorption was stronger for aromatic solvent-cast MEH-PPV films at 633 nm, which leaded to more absorption losses.

The distinct morphologies and ASE performances confirm the conformation difference when MEH-PPV films are spin-cast with different solvents. The conformations show intimate contact with the solvent polarity and molecular structure. Aromatic weak polarity solvents such as CB, toluene, and p-xylene have a preferential interaction with the aromatic backbone of the MEH-PPV polymer chains, and thus the chains possess a rigid and open conformation in solution. As a result, it is straightforward for the *π*-electrons on one chain to overlap with those on an adjacent chain when spin-cast, e.g., the conformation shows intense aggregation, which corresponds to higher average surface roughness, ASE threshold, waveguide losses, and lower net gain properties. Nonaromatic strong polarity solvents such as THF and TCM, on the other hand, have a preferential interaction with the polymer’s side groups. Thus, the polymer chains in THF and TCM coil tightly to maximize solvent-side group interactions and minimize exposure of the aromatic backbone to the solvent. Thus, the conformation possesses weak π-electron interactions with adjacent polymer chains when spin-cast, e.g., weak aggregation [11,21,22], which corresponds to lower average surface roughness, ASE threshold, waveguide losses and higher net gain properties. The volatility for non-aromatic strong polarity THF and TCM occurs faster than that of aromatic weak polarity CB, toluene and p-xylene, which provides the possibility for maintaining the conformation well when spin-cast, as shown in Table 1 [29].

The major reason for the distinct ASE performances is the presence of exciton annihilation, which is described by a model expressed as [11]

(5) $$\frac{dN(t)}{dt}=-\frac{N(t)}{\tau }-\frac{\beta }{\sqrt{t}}N^{2}(t),$$

where *N*(*t*) is the time-dependent population density of emissive excitons, *τ* is the exciton life time, and *β* is the bimolecular recombination coefficient. The exciton life time is essentially identical, while the bimolecular recombination coefficient is nearly an order of magnitude larger in the aromatic weak polarity solvent-cast films than in the nonaromatic strong polarity solvent-cast films [11]. The excitons migrate to adjacent polymer chains when exciting. As a result, the exciton annihilation occurs and quenches the luminescence. The exciton annihilation is morphology-dependent, e.g., the exciton annihilation increases with aggregation. For MEH-PPV films spin-cast with nonaromatic strong polarity solvents such as THF and TCM, the morphology of the polymer chains shows weak aggregation. As a result, the exciton annihilation is suppressed in comparison with the MEH-PPV films spin-cast with aromatic weak solvents such as CB, toluene and p-xylene. Thus, it is not surprising that THF- and TCM-cast MEH-PPV films possess a lower ASE threshold and a higher net gain.

### 3.4. Pure Film Spectra Characterization

**Figure 6:**
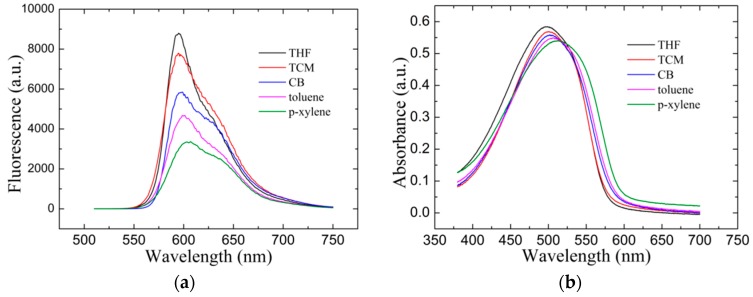
Pure MEH-PPV film spectra characterization: (**a**) fluorescence spectra and (**b**) absorbance spectra of the spin-cast MEH-PPV films from different solvents.

The spectroscopic characterization was performed to further confirm the distinct conformation of the spin-cast MEH-PPV films [11,21]. The thickness of the MEH-PPV films were controlled at 80 nm. Figure 6a shows fluorescence spectra of the pure MEH-PPV films spin-cast from THF, TCM, CB, DCM, toluene and p-xylene. The reabsorption of fluorescence emision was inhibited because of the large stokes shift (~100 nm) in comparison with the absorption spectra as shown in Figure 6b [3]. The results showed that the fluorescence spectra were distinct for shapes and peak locations of the spectra. The peaks of the fluorescence spectra represented characteristic vibronic structure. The singlet S_0-0_ peak located at 594.2, 594.8, 597.8, 600 and 604 nm for THF, TCM, CB, toluene and p-xylene spin-cast films, respectively. The fluorescence peaks for aromatic weak polartity spin-cast MEH-PPV films were red-shifted in comparison with that spin-cast with non-aromatic strong polarity solvents [11]. The reason is the increasing of conjugation length for aromatic weak polartity spin-cast MEH-PPV films [22]. The fluorescence emission intensity decreased evidently for aromatic weak polartity spin-cast MEH-PPV films in comparison with that spin-cast with non-aromatic strong polarity solvents.

Figure 6b shows absorbance spectra of the pure MEH-PPV films spin-cast from THF, TCM, CB, toluene and p-xylene. The full width at half maximum (FWHM) of all the absorbance spectra were over 100 nm. The absorbance peak located at 498, 499.8, 502.3, 505.9 and 512.8 nm for THF, TCM, CB, toluene and p-xylene spin-cast films, as shown in Figure 6b, respectively. The absorbance peaks for aromatic weak polartity spin-cast MEH-PPV films were red-shifted in comparison with that spin-cast with non-aromatic strong polarity solvents. The reason for this is the increasing of the conjugation length for aromatic weak polartity spin-cast MEH-PPV films [22]. The distinct spectral shapes and peak locations of the fluorescence and absorbance indicates the disctinct energy levels of the conformations. We believe that the difference of fluorescence and absorbance spectra was due to distinct confromation of the spin-cast films.

### 3.5. Lasing Properties

**Figure 7:**
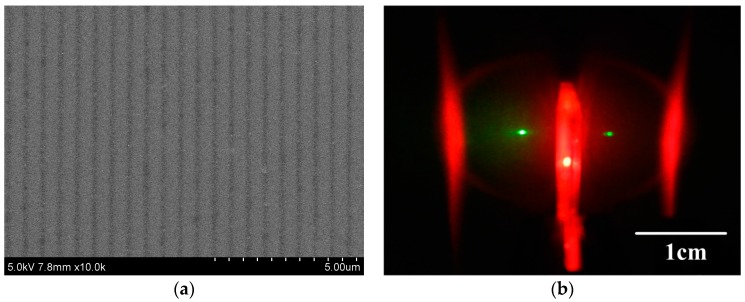
(**a**) Scanning electron microscope (SEM) image of the HPDLC film and (**b**) emission beams pattern of the waveguide HPDLC DFB laser.

For laser applicaton, a HPDLC grating was fabricated on the MEH-PPV film as the external light distributed feedback geometry to form HPDLC/MEH-PPV/glass asymetric waveguide. According to Kogelnik’s formula of the theory of DFB lasers [30], the lasing emission wavelength in vacuum is

(6) $$\lambda_{las}=\frac{2n_{eff}\Lambda }{m},$$

where *n_eff_* is the effective refractive index of the lasing mode, *Λ* is the period of the grating, and *m* is the diffraction order. The lasing wavelength can be tuned by changing the grating period Λ and the effective refractive index *n_eff_*. The thickness of the films were controlled at 80 nm. For the diffraction order the 3rd, taking the value of 1.609 for effective refractive index into account, we made HPDLC grating with 590 nm period to obtain the output lasing wavelength around 633.4 nm. The lasing, which is coupled out via grating coupling [20,31,32,33], as shown in Figure 1d and Figure 7a, shows the SEM image of the 594 nm period HPDLC grating, which confirms the well-defined and uniform grating configuration. The fan-shape-like emission beams pattern is illustrated in Figure 7b, which confirms the good directionality of the HPDLC DFB laser in comparison with the ASE emission pattern in Figure 4c.

**Figure 8:**
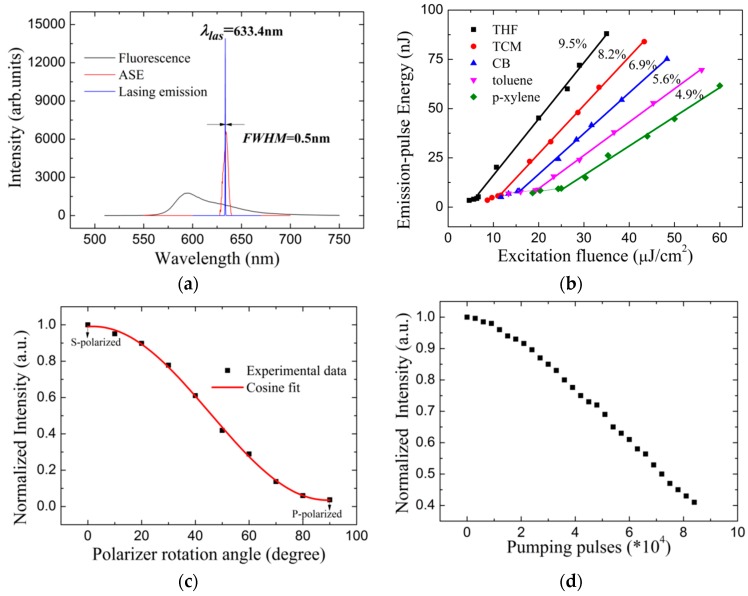
Lasing emission properties characterization: (**a**) lasing spectrum of THF-cast sample gathered at an excitation fluence of 83 μJ/cm^2^; (**b**) dependence of emission-pulse energy on excitation fluence; (**c**) normalized emission intensity as a function of polarizer rotation angle, and; (**d**) dependence of normalized emission intensity to pumping pulses for THF-cast laser.

Figure 8a shows the lasing spectrum from THF-cast MEH-PPV laser at excitation fluence of 83 μJ/cm^2^. The central wavelength of the lasing spectrum was 633.4 nm, which was consistent with the theory described by Equation (6), and the full width at half maximum (FWHM) of the lasing spectrum was 0.5 nm. The spectral compression and selection was excellent for the lasing emission spectrum of the waveguide HPDLC DFB laser in comparison with the fluorescence and ASE spectra. Figure 8b shows the emission-pulse energy as a function of excitation-pulse energy. The emission energy increases slowly with the excitation energy at the initial stage, and then the emission energy increases abruptly when the excitation energy reach the lasing threshold as shown in Figure 8b. The lasing threshold for THF, TCM, CB, toluene and p-xylene spin-cast waveguide HPDLC DFB laser was 6.7, 11.7, 15.7, 20 and 25 μJ/cm^2^, respectively. The slope conversion efficiency of input pulse energy to output pulse energy for THF, TCM, CB, toluene and p-xylene cast HPDLC DFB laser was 9.5%, 8.2%, 6.9%, 5.6% and 4.9%, respectively. It indicates that the conformation of the spin-cast MEH-PPV films correspondingly make a difference to the performance of laser devices. The lasing threshold tends to increase with aggregation. However, the conversion efficiency decreases with aggregation. The reason is that the aggregation leads to bi-molecule non-radiative emission process, which quenches the florescence [34,35,36]. The emission beams showed excellent s-polarization as shown in Figure 8c. The device lifetime [37,38,39], which defined as the numbers emission pulses when the emission energy drops to half of the initial intensity, was about 72,000 pulses after 2 h 10 Hz pumping with the excitation fluence at 1 mJ/cm^2^, as shown in Figure 8d. The lifetime confirms the photo-stability of the laser device. From our study, the indication is that the conformation with weak aggregation is a good candidate to be used as the laser active medium.

## 4. Conclusions

In conclusion, the morphology and aggregation was systematically investigated for the lasing emission of semiconducting polymer poly[2-methoxy-5-(2′-ethyl-hexyloxy)-1,4-phenylene-vinylene] (MEH-PPV) films with aromatic weak polarity solvents chlorobenzene (CB), toluene, p-xylene and non-aromatic strong polarity solvents tetrahydrofuran (THF) and trichloromethane (TCM). The results indicated that the conformation of the spin-cast MEH-PPV films with weak aggregation performed excellent lasing emission performances because of the inhibition of the fluorescence quenching induced by the bi-molecule process. The Atomic Force Microscope (AFM) images showed morphologies with different average surface roughness. The amplified spontaneous emission (ASE) experiments confirmed the distinct polymer chain conformation. The conformation, which preserved from the spin-cast process, indicated the distinct interactions between the solvents and MEH-PPV polymer chains. The distinct conformation leads to different interactions of the π-electrons on one chromophore to neighboring polymer chains. The pure film spectra were performed to confirm the effect of distinct conformation on the energy level. This study provides insight into the morphology and aggregation effect of the spin-cast polymer films on the performances of lasers.

## Figures and Tables

**Table 1:** Properties of solvents used in this work. Tetrahydrofuran (THF); trichloromethane (TCM); chlorobenzene (CB).

Materials	Chemical Structure	Polarity	Volatility (mg/hour)
THF	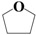	4.2	687
TCM	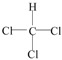	4.4	323
CB	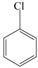	2.7	43
toluene	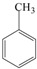	2.4	79
p-xylene	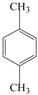	2.5	27
